# LGBTQ AGING PARADOX: NEEDING WHILE AVOIDING LONG-TERM SERVICES AND SUPPORTS

**DOI:** 10.1093/geroni/igad104.1362

**Published:** 2023-12-21

**Authors:** Rajean Moone

**Affiliations:** University of Minnesota, Minneapolis, Minnesota, United States

## Abstract

A growing body of literature recognizes the significant health disparities experienced by sexual and gender minority (SGM) older adults including higher rates of chronic conditions, mental illness, and disabilities in comparison to their peers. Simultaneously SGM older adults are more likely to age alone and not have a friend or family caregiver to provide support. At the same time SGM older adults express hesitation and fear in accessing formal long term services and supports (LTSS). One study of trans older adults noted that suicide was a preferred option to a nursing home. This paper will outline a complex paradox: Many SGM older adults (1) experience significant health disparities which may require additional support; (2) lack informal support thus needing to rely on formal LTSS; and (3) avoid formal services due in large part to fear of discrimination and poor treatment. Opportunities to mitigate the paradox through education and awareness as well as implications for policy and research will be discussed.

